# Digital platforms: Perceived criteria of success, importance of work design, occupational safety and health for present and prospective digital platforms

**DOI:** 10.3233/WOR-211253

**Published:** 2022-08-11

**Authors:** Katharina Schäfer, Arne Görke, Luis Hesemann, Tim Franke, Verena Nitsch, Christoph Heckwolf, Alexander Mertens, Christopher Brandl, Axel Zweck

**Affiliations:** a Industrial Engineering and Ergonomics, RWTH Aachen University, Aachen, Germany; b Innovation und Entrepreneurship (WIN), RWTH Aachen University, Aachen, Germany; c Innovation and Future Research, Institute of Sociology, RWTH Aachen University, Aachen, Germany; d Fraunhofer Institute for Communication, Information Processing and Ergonomics FKIE, Aachen, Germany

**Keywords:** Platform-based labour, OSH, future research, comparative analysis, structured content analysis

## Abstract

**BACKGROUND:**

Digital platforms have found their way into all our lives: they are discussed in political, economic, scientific and public fields worldwide. Platform-based work is also on the rise in the German labour market, not only in institutionalised work, but also in start-ups and spin-offs.

**OBJECTIVES:**

The article describes the results of an analysis aimed at identifying perceptions of new and already known major success factors on market entry and market penetration regarding occupational safety and health (OSH) and work design.

**METHODS:**

A total of 31 semi-standardised interviews were conducted with 39 people. First, perceived success factors in general were examined with the comparative analysis. Surprisingly, OSH/work design factors did not emerge as perceived success factors. For this reason, a in-depth analysis was performed in a secondary analysis with the structured content analysis.

**RESULTS:**

Identified perceived success factors were user orientation, scalability, network effects, niche occupation. The in-depth secondary analysis with focus on OSH/work design showed that the interviewees are aware of the topic of OSH/work design, but did not consider it to be important to economic success.

**CONCLUSIONS:**

The identified success factors may not seem surprising. What is surprising, however, is the role played by OSH/work design. Solutions must be developed that sensitize working persons in the platform sector to the topic of OSH/work design. A two-step process may be useful: First, uniform regulations and laws must be anchored in the platform architecture. Second, various measures and training courses can be designed to inform and raise awareness.

## 1 Introduction

Digital platforms were introduced at the beginning of the 2000s. Nowadays, it is almost impossible to imagine daily life without them. Digital platforms shape our vacation habits (e.g. AirBnB), influence our mobility options (e.g. Uber) and modify our communication (e.g. Facebook, Snapchat) [1–4]. Due to digitisation processes, platforms are receiving increased political, economic, scientific and public attention worldwide [5]. When it comes to work and labour processes, various activities can be outsourced on crowdwork platforms. The current social developments caused by the Coronavirus Crisis and the resulting implications are expected to give a further boost to digitisation and remote workforms for some jobs. If so, this would have a direct impact on labour, whether platform-based or institutionalised. These changes could be of structural, institutional or organisational nature. In this context, the term “online labour” is a collective term for a far-reaching phenomenon that includes, for example, the gig economy, platform-based work and crowdwork [6]. It can be assumed that within this digitisation boost not only further socio-technical infrastructures will emerge, but at the same time will also greatly shape our perceptions of work in the near and distant future. The current development also

“provides a new wave of entrepreneurs and businesses with opportunities to innovate based on their creative skills and knowledge in areas such as product design, virtual tourism, spaces and environment aesthetics, social exchange and simulations” [7]. 

Consequently, it is reasonable to assume that as the number of employees in the area grows, discussions about work design, occupational safety, and employee health will become more important in the future [8]. Taking a closer look at the current situation of entrepreneurs as well as people working on platforms highlights many risk factors and obstacles, such as financial insecurity, conflict of interest, large responsibility, high number of working hours, managing tasks, and administrative burden [9, 10]. Smaller companies in particular face the problem that occupational safety and health (OSH) is not sufficiently taken into account [11]. The increasingly digital components and the associated predominantly virtual communication represent an additional obstacle [12]. But even with hybrid work models, such as online delivery services, there are already studies that suggest that there is a risk of injury to the deliverer and that corresponding preventive measures are necessary [13]. Accordingly, platform-based work is currently being discussed controversially. It is stated that more research is needed in the various areas that deals with the topic [14]. An identification of relevant factors at this stage could help to create a foundation for future platform-based work- and labour processes, particularly to successfully establish and institutionalise platform-based work in Germany, but also internationally. Here it is assumed that OSH as well as good work design, fair working conditions, ergonomic standards will have to be given high priority by the stakeholder in the newly establishing field in order to be able to establish platform work in society –or to prevail against other platforms on the market. Consequently, ergonomic standards must also be applied to each of these activities.

Based on these considerations, the following research question has been derived: (Q1) What are the potential success factors of platform-based work of start-ups in Germany? Among other aspects, we focus on the identification of perceived and thus subjective new and previously identified success factors: market entry and market penetration, current and future implications as well as the chances of organisational growth associated with these criteria. A qualitative approach was chosen in order to be able to identify potential new factors. An analysis based on the comparative analysis [15], utilised the categories and dimensions identified by Engelhardt et al. [16], who conducted a Germany-wide study with managers, resulting in specific characteristics and success factors for digital platforms, as a starting point. Engelhardt et al. thus conducted 14 structured telephone interviews with managers in Germany. The approach here was to check the results with a larger sample on the one hand and to deepen them on the other. A more diverse sample was chosen (founders, platform employees, citizens and experts) to counteract potential sampling effects. Thoughts on current and future development potentials refer to future and innovation research and ergonomics [17–20]. Although the field of platform-based work is controversial and a number of risk factors regarding OSH and work design have already been identified in the current literature, this was not amongst the named success factors [9–14]. For this reason, a second research question was posed: (Q2) What do the interviewed people think about OSH/work design as a potential success factor? An in-depth secondary analysis with the structured content analysis was conducted that focused specifically on the perception of OSH/work design as potential success factor [21, 22]. Accordingly, the contribution first presents the results of the primary analysis of the success factors market entry (Q1) and market penetration and afterwards addresses the results of the secondary analysis of the OSH/work design criteria (Q2).

## 2 Methodology

### 2.1 Questionnaire construction

The semi-standardised interviews were designed with a total of nine key-questions, each addressing different categories, such as the organisational structure of platforms, reasons for success or failure of platforms, distinguishing features of platforms, legal/social/ethical/ergonomic conditions of platforms (see Table 1) [23–27]. Deviation from the guideline was possible at any time. During the interview, each key question was asked to all interviewees. Thematic follow-up questions were asked afterwards. If the conversation stalls specific questions were raised in individual cases. The goal was to answer the main questions without being too prescriptive.

**Table 1:** Questionnaire construction

Key question (Initial question)	Checklist –Has this been mentioned? (Only inquiries, if not addressed by itself)	Examples of specific questions (In appropriate position)
(1) I would like to know how your platform or start-up was created	• Challenges	• What were your aims?
	• Reasons/motives for spin-offs	• What are the challenges in the development and conception as well as in the initial period of the platforms/start-ups?
	• Aims of the platform	• ...
	• ...
(2) Can you tell us something about the structures within your company / platform / start-up?	• Perception of the division of labour	• How do you divide the tasks?
	• Desire in the division of tasks	• Do you source out certain tasks?
	• Conditions for the division of labour	• ...
	• ...
(3) What are the reasons for the success of your platform?	• Subjective sensing of the respondent	• Occupation of market/technological niches?
	• Objective criteria	• Expansion as a recipe for success?
	• Wishes	• ...
	• Expectations
	• ...
(4) What are potential reasons for the failure of (/ your) platforms / start-ups?	• Subjective sensing of the respondent	• In your opinion, what factors are essential when platforms/start-ups fail?
	• Objective criteria	• Does the network of stakeholders play a role when platforms fail?
	• Wishes	• ...
	• Expectations
	• ...
(5) How does your digital platform differ from other platforms?	• Regarding opportunities and problems	• Do you observe international platforms, or do you compare yourself with them?
	• From a social point of view	• Is your platform internationally oriented? Do you operate internationally? (data, users, employment relationships)
	• In developing platforms	• ...
	• During the first time/phase
	• Compared to international platforms
	• ...
(6) What role do legal/ethical/social frameworks play for digital platforms?	• Specific conditions	• What relevance does data handling have for you?
	• Employee rights	• To what extent do legal/social/ethical conditions for data handling (storage, sharing, monetization of data) play a role for you?
	• Data	• ...
	• National and International
	• Risks and \challenges
(7) What impact do technological developments have on digital platforms, or start ups?	• Opportunities	• Which technological developments are currently relevant; which could become relevant in the future?
	• Risks and challenges	• Potential fields of application of new technologies for digital platform systems?
	• Knowledge/Know how	• ...
	• Structure/platform architecture
	• In 5 years
	• In 10 years
(8) What do you think is changing in society as a result of these (digital?) forms of work?	• In the next 5 years	• Do you think there will be a change in our understanding of work?
	• In the next 10 years	• What might such changes look like?
	• Regarding the concept of work	• ...
	• What is expected
	• What is desired
	• ...
(9) How do you estimate the development of the work domain until 2030?	• Routine tasks	• Do you think that will change?
	• Creation of new jobs	• Where do you suspect changes beyond that?
	• Salary	• ...
	• Positive and negative effects
	• Effect of technology
	• Work location+working hours
	• ...

### 2.2 Restriction

The present study has several restrictions. Firstly, two different interviewers conducted the interviews, secondly, the interviews took place in different locations. Due to various participant locations within Germany, interviewers visited partakers if possible. If this was not possible, the interviews were conducted online via “Skype”. A further restriction is the selection of stakeholders: speaking of platform-based work, there is –depending on the sector –a multitude of stakeholders who play a major role. Additionally, different demographic characteristics such as age, gender, origin, social status etc. are not equally present in the study [28].

### 2.3 Procedure

**Fig. 1:**
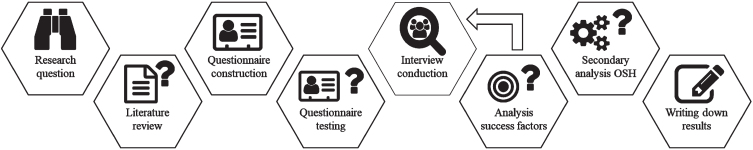
Procedure of the research.

**Fig. 2:**
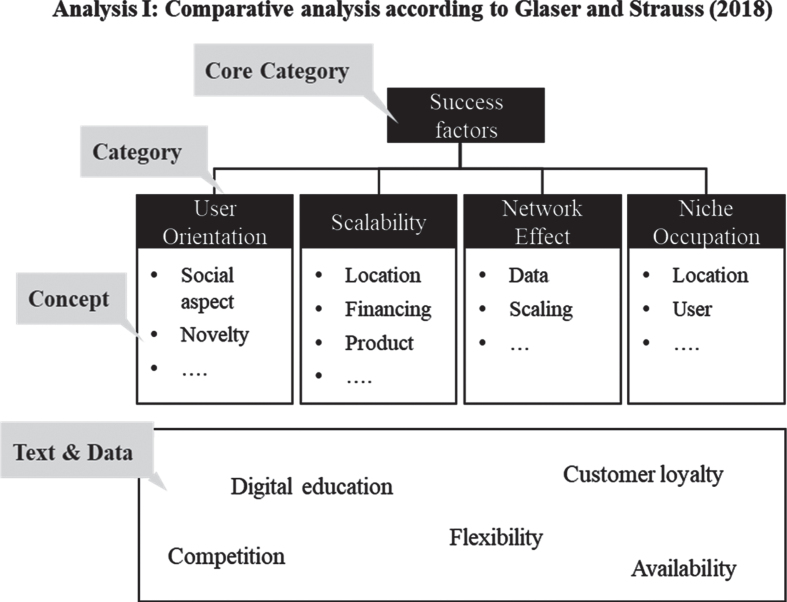
Example of comparative analysis.

The data was collected from June to October 2019, consisting of 31 semi-standardised interviews, conducted with 39 people (see Fig. 1). The participants of the interviews were not paid. In addition, single and pair interviews were carried out [29]. All interviews were conducted in German to ensure general comprehensibility. The duration of the interviews varied from 40 minutes to up to two hours, each of them starting with a general introduction and the guidelines of qualitative interviewing, followed by the privacy policy. Afterwards, a short questionnaire on demography was filled out by the participants before the actual interview took place. The audio files of the interviews were transcribed by an external provider and analysed with the MAXQDA software. The analysis for Q1 was based on the comparative analysis [15, 30–32], in which patterns are reconstructed. The analysis followed a multi-stage process. First, the entire data was skimmed and divided into sections. This was followed by an in-depth analysis in which concepts were extracted. These were then clustered into categories and finally summarised under the main category of “success factors” (see Fig.  2).

**Fig. 3:**
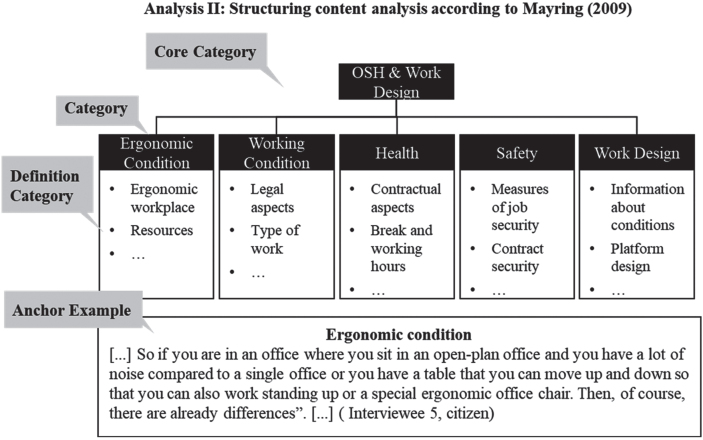
Example of structuring analysis.

The secondary analysis for Q2 was carried out by means of content analysis [22]. For this purpose, the interviews were analysed in a second run. In addition to the theory-based derivation of the category system, the categories were also defined. Furthermore, anchor examples were determined and the entire material was analysed several times (see Fig.  3).

### 2.4 Participants

**Fig. 4:**
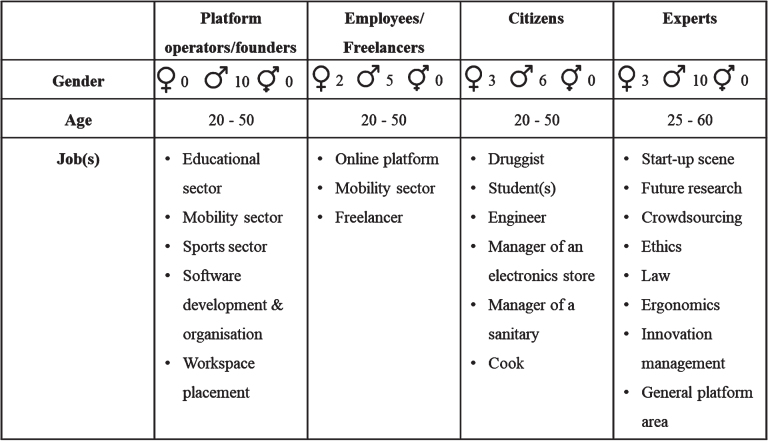
Demographic information of the interviewees.

Prior to the actual empirical work, relevant stakeholders who play a major role in platforms were identified. It is assumed that stakeholders in the platform sector interact within a dynamic structure [33]. Descriptive features can be seen in Fig.  4. Though the change of positions is fluid, the four stakeholder positions below can be distinguished from one another. The four groups were divided as follows:

(I) Platform operators/founders: Operators and founders are located at the management level of platforms. The perspective of the superiors and founders should be recorded, including CTOs, CEOs, department directors and other key players at the management level. This group was identified as an important stakeholder due to their leading role in the design of fair working conditions and their knowledge on platform structures [34, 35].
(II) Employees/Freelancers: to complement the perspective of the management level, employees and freelancers were also identified as a stakeholder-group. It is assumed that employees/freelancers are often more affected by risks related to, for example, platform-based labour [36, 37].
(III) Citizens: For the overall social analysis, citizens who had direct or indirect contact with digital platforms were also surveyed. As potential platform users, their perspective, knowledge and expectations of platforms ere investigated. At the same time, the aim was to involve citizens in scientific work in the sense of participation [38]. Citizens who use or work with platforms are directly affected by the implications of these digital infrastructures, but their perspective is all too rarely considered.
(IV) Experts: Finally, experts in the field of platform-based work were interviewed [39, 40]. This complemented the perspectives on the current opportunities and challenges and the ongoing development in the platform sector.

## 3 Results

First the subjective stakeholder perceptions of the success factors are presented: Chapters 3.1 –3.4 present the results of the comparative analysis for answering Q1. Chapter 3.5 describes the results of the OSH/work design factors of the structuring content analysis to answer Q2.

### 3.1 User orientation

The interviewees characterized “user orientation” by the following features: User orientation can refer to the offer, product or service of the platform (see Table 2). It must fulfil the wishes, needs and requirements of the users and aim to build an opportunity, so that the user is able to identify him-/herself with the platform or product offered (Interviewee 27, citizen). A demand is generated within satisfying the wishes, needs and requirements, even “if perhaps the user does not know yet that he has that demand” (Interviewee 23, employee,). On the one hand, the identification of needs plays an important role; while on the other hand, the platform had to evoke a relationship with the user. An important factor here is a basis of trust that can be created by, for example, free partial services. Additionally, platforms should have unique selling propositions for their product/service, since user groups are often “flooded with offers” (Interviewee 27, citizen).

**Table 2:** Overview of mentions in user orientation sorted by stakeholder

	Platform operators/founders	Employees/Freelancers	Citizens	Experts
Product/service		•	•	•
Social aspects	•		•	•
Novelty			•	•
Usability	•		•	•
Data	•	•	•

Other named criteria for platforms were problem reduction (in terms of individual needs during use) and increased (emotional) value of the product/service (Interviewee 8, expert). Looking at social aspects, feedback was named as one main criterion: By obtaining feedback, the user’s needs can be met more precisely, which further strengthens identification with the platform or product (Interviewee 26, citizen; Interviewee 6, founder). Another important criterion named by one expert, was the possibility of product recommendation (Interviewee 25, expert). Further, the integration of the user in the process of (product-) development is important for success of platforms. Inclusion can take place through interaction, which in turn can lead to an increase in user confidence.

Another important aspect for user orientation was a contemporary range of products and services and thus the degree of novelty: “You really have to be up to date, always keep up with the times and still run the risk of some new platform coming along [...]” (Interviewee 27, citizen). However, in the context of contemporary offerings, it is important to retain users in the long term (Interviewee 27, citizen). Convenience as a criterion for success may be helpful here (for example by making the process of shopping as simple and clear as possible) (Interviewee 29, expert; Interviewee 3, founder).

In addition to the satisfaction of needs and novelty of the service/product, usability criteria played an essential role for successful platforms. These criteria included user-friendliness, a easy-to-use interface or even transparency. Transparency referred to the compatibility of user goals with the platform’s offerings (Interviewee 16, expert). In addition to an attractive user interface, low costs or ease of use can increase user acceptance (Interviewee 10, citizen). Other measures worth considering were the integration of functions from other platforms followed by user convenience (Interviewee 4, founder).The way in which data is collected, managed, stored and passed on is a further criterion for success. Through the exchange of information between user and operator, a basis of trust can be created, which ensures long-term use of the platform. “But if you do not fix it, [...] the user will not be able to see the big vision. Because he gets distracted by so many other things, and then he does not trust you as a platform” (Interviewee 4, founder). The recording of data (e.g. also metadata, key performance) was also important to understand the user’s behaviour in digital environments (Interviewee 23, employee; Interviewee 8, expert). This allows unfiltered information to be collected and ensures the identification of critical attitudes that are not actively communicated. According to one expert, platforms, such as Amazon tried to facilitate market overviews for private users and highlight certain products for which they received a service from the manufacturer in return (Interviewee 18, expert). Socially, however, this is viewed critically.

### 3.2 Scalability

Scalability was a success criterion, especially under the condition of global aspects: “By the way, I would also describe the possibility of getting in touch with people from all over the world as one of the success factors” (Interviewee 19, employee) (see Table 3). Scalability thus refers to the global effect of networking and thus equally refers to network effects. Scalability can lead to success in the context of expansion –both national and international. To achieve critical mass, one can “[...] make the platform more valuable because it has more participants [...]” (Interviewee 8, expert). Another aspect of success mentioned relates to financing. Platforms appeared attractive for investors thanks to their scalability, which could possibly be converted within reach in terms of new users. “You have this idea. You raise a lot of money through investors with this idea. And afterwards you try to scale as high as possible and establish yourself directly in the market” (Interviewee 12, founder). As user numbers increase, digital platform gain two advantages: First, the increasing number of users is accompanied by no great technological or economic effort. Secondly, the service of the platform can be optimized due to increasing user numbers and the associated user data.

**Table 3:** Overview of mentions in scalability sorted by stakeholder

	Platform operators/founders	Employees/Freelancers	Citizens	Experts
Location		•		•
Financing	•
Product/Service	•		•
Social aspect		•
Direct networkeffect			•	•

A further scaling aspect was the acquisition of market shares: “And with a platform [...] or a good product, of course, you can achieve a lot of market segments relatively efficiently” (depending on the size of the platform) (Interviewee 10, citizen). It is equally important to distinguish whether the platform is a mere software solution or whether it includes physical components. With the former, an increase in the number of users is comparatively easy (Interviewee 12, founder). For the latter there are fixed costs if scaling is sought due to defects or failures, for example in the mobility sector around platforms (Interviewee 12, founder). For a software solution it is crucial to implement the technical option to scale as soon as possible and furthermore always keep scaling in mind while developing new features (Interviewee 12, founder). Especially when platforms are about to enter the market and have few financial resources available, maximum output must be generated with as little effort as possible (Interviewee 12, founder; Interviewee 5, expert.).

The idea of social aspects and thus social networking is often equated with platforms and is an important part of scaling. In order to achieve scaling, one can “[...] first create a basis, gain experience and then develop and expand (Interviewee 23, employee). This social phenomenon can be observed in direct network effects. At best, the option to reach as many other users as possible allows the user base to grow and –in doing so –results in a win-win situation for users and developers (Interviewee 28, citizen). An increased traffic by an increased user base might be one positive consequence, which in turn increases the added value and makes the platform more attractive to its users. Particularly challenging in this context are the risks of takeover and the copying of main features by large companies. This can also be the goal of small platforms/start-ups. However, protection against these substitutions represents a high barrier for market-entry. On the one hand, this is due to the high investment costs of an offer which is difficult to copy. On the other hand, barriers for market-entry arise from the attainment of a critical user mass and the resulting network effects (Interviewee 13, expert).

### 3.3 Network effects

The social networking, i.e. transactions and interactions between users within digital platforms was thus becoming a key factor in ensuring the success of the platform (Interviewee 30, citizen) (see Table 4). Networking creates additional opportunities for the user: “Now, for example, on Facebook, the more people are around, the more events you have. Or at Amazon, the more customers there are and the more salesmen there are, the greater the range you can offer” (Interviewee 31, citizen). Rapid scaling due to network effects and the associated possibility of convincing investors is one way of success looking at network effects (Interviewee 5, employee; Interviewee 19, employee). A further aspect of network effects was that they occur as a self-reinforcing system: “In other words, network effects in the sense of synergies and reinforcement of ideas, i.e. input, output” (Interviewee 16, expert). According to experts, a negative result of network effects was the need for access regarding individual platforms and users: If many users are drawn to a platform and only communicate with others via this platform, people who do not use it could be excluded (Interviewee 16, expert).

**Table 4:** Overview of mentions in network effects sorted by stakeholder

	Platform operators/founders	Employees/Freelancers	Citizens	Experts
Direct networkeffect		•	•	•
Indirect	•		•	•
networkeffects
User orientation	•		•	•
Scaling		•
Data	•

Indirect network effects can equally lead to success: Conversely, the more offerings a platform has, the more attractive the platform is when it comes to generating a demand of side-users. Clearly, it implies a high level of user satisfaction, which supports the network effects. In order to ensure user satisfaction, the platform must be user-oriented, and its needs have to be addressed (Interviewee 27, citizen). Furthermore, it is possible to implement additional cross-platform functions through cooperation with other platforms in order to benefit from each other’s popularity (Interviewee 13, expert). As a result of successful cooperation’s, new companies become aware of the platform. Particularly in the case of multi-sided platforms, these (in-/direct) network effects allow both the supply side and the demand side to grow independently of each other. Indirect network effects then strengthen the growth beyond this point (Interviewee 12, founder).

Network effects seem to be closely linked to user orientation: “As I said, everything becomes easier for the users. That is why they also use the platform, because it is easy to get in contact with others, i.e. business partners” (Interviewee 28, citizen). A platform should first gain user acceptance before network effects can take place. Afterwards, direct network effects could lead to “[...] organic, exponential growth, which you could never build up through sales” (Interviewee 3, founder). Furthermore, platforms can gain popularity by cooperating with “influencers”. Influencers can be defined as persons who, for example, disseminate information through social networks [42]. These influencers can act as role models for other users in favour of the platform and increase the platform’s traffic through their actions. In addition, cooperation with influencers from other platforms, especially in the social media sector, would be an opportunity to benefit from their reach and to convince potential users to join (Interviewee 16, expert).

With regard to software products, scaling should also be mentioned here as a prerequisite for the success factor of network effects. Software products in particular, can be easily scaled up and become widely used in the age of digitalisation in some instances. By means of an application via cloud services, it is –under certain circumstances –possible to track actions of the user. These activities draw better conclusions regarding the needs of the user and their interaction. It is also possible to infer conclusions about the relationship between users and to trace the first platform contact (Interviewee 5, employee). In the best case, this favours scaling (Interviewee 14, employee).

A further advantage that was mentioned was an increased acquisition of data in the context of network effects (Interviewee 12, founder). Thereby an evaluation of the available data for measures of scaling is necessary: “That means, we must ACTIVELY look at ourselves at what happens in these courses, how humans behave and then have to convert this also” (Interviewee 12, founder).

### 3.4 Niche occupation

Niches, broadly understood as regional and exclusive niches, are another success factor (see Table 5). An important criterion mentioned in the interviews was the size of the corresponding niche:

**Table 5:** Overview of mentions in niche occupation sorted by stakeholder

	Platform operators/founders	Employees/Freelancers	Citizens	Experts
Location			•	•
User		•
Market entry	•	•
Financing	•			•
Platform offers		•

“[...]So it is now a platform that you serve with a niche that can now work globally or is something that really only works locally and I think if it only works locally it is probably not a viable business model because then the niche is just too small. And even if it works for two years, the point of view simply cannot grow anymore. But if it is something that you can expand globally or even further, or from the niche somehow out of this niche, then develop a broader concept, then of course it can work” (Interviewee 28, citizen).

Nevertheless, the objective of platform-based business models is to scale up and grow beyond the niche (Interviewee 15, expert,). Regional platforms are only advantageous if they address a precisely identifiable user group, because they have a small reach but can cover their niche more effectively (Interviewee 22, expert). In addition, an expert states that established platforms are targeting niches (Interviewee 22, expert). Regarding increased economic and creative competition, large companies can also use their capital for takeovers. Even established platforms can serve a niche by adding a feature. As limiting the user group can support platforms to build up a unique selling point and thus become more attractive (Interviewee 20, employee). This results in a stronger individuality, which also plays a major role in the context of niche occupation. By limiting the platform to a local market and specifying services offered to small user groups, they contribute to success in niches. As mentioned another important criterion was the size of the niche. It should always be identified and evaluated beforehand: “Just because it is a niche does not automatically mean that there is success behind it. Because a niche can be small and therefore somewhere not profitable for the platform” (Interviewee 23, employee). Often, however, a niche cannot be calculated precisely, so that occupying an unpredictable niche represents a high risk, especially for established companies (Interviewee 23, employee).

Some of the founders/platform operators assessed the phenomenon of niche occupation much more critically than citizens and employees/freelancers. Reasons were the limited user base and thus the lack of exponential success (Interviewee 3, founder). Especially exponential growth was important as a criterion for the platform operators/founders. If this is not given, they saw reduced chances of success. At the same time, a few founders saw the occupation of niches as the only possibility for new platforms to enter the market, as otherwise, the demand based on users’ needs was missing and the competition from large market participants leads to major barriers (Interviewee 1, founder). Comparing a platform that occupies a niche to small or medium-sized enterprises/companies, some common features might appear (e.g. flexible structures in start-ups), but they differ fundamentally in terms of long term perspectives. The platform aims to scale and expand out of the niche, while the medium-sized company can grow, but does not have the network effects and low transaction costs of digital platforms (Interviewee 3, founder).

Of special importance to the subject of niche occupation is the required starting capital for entering a niche, which is acquired by investors. If a niche is too small and thus cannot ensure sufficient profitability, it is difficult to convince the investors. One possibility mentioned was the conception of a platform that occupies niches and has further incentives (Interviewee 3, founder). While the mass market is dominated by established companies, niches have a much smaller potential user base and thus a comparatively low business volume. Nevertheless, a niche specialization can be profitable for a digital platform due to the lack of competition. Thus, it is possible to acquire users in the niche through a secured market position –presumed the critical mass is reached –and to exploit network effects (Interviewee 18, expert).

Occupying technological niches was another criterion for the success of platforms. According to one interviewee (Interviewee 19, employee), an expansion of successful niches was quite possible through network effects, but does not necessarily has to take place.

### 3.5 OSH/work design factors in the platform area

In the case of work design, only one employee stated that information on the design of contracts could be found on the Internet (Interviewee 5, employee) (see Table 6). A total of two founders commented in a generic way: In their opinion, platforms have to be designed fairly for users and workers and companies need freedom to expand (Interviewee 4, founder; Interviewee 2 founder). Among the experts, one said that there should also be regulations on data protection with regard to freedom from harm and interference and that these should be observed (Interviewee 16, expert). Another expert stated that the collective actor (i.e. social groups) will become relevant for the future world of work and its design (Interviewee 22, expert).

**Table 6:** Overview of mentions in OSH/work design factors sorted by stakeholder

	Platform operators/founders	Employees/Freelancers	Citizens	Experts
Work design	•	•		•
Ergonomic condition		•		•
Working conditions	•	•	•	•
Health		•		•
Safety		•	•	•

A similar picture emerges with regard to ergonomic conditions: One employee stated that there are no ergonomic workplaces, but that there was a chair and a table and that the conditions were therefore comparable to common office workplaces (Interviewee 5, employee). One expert stated that there were already defined standards for ergonomic workplace design and that he did not believe that further rules and regulations would be beneficial at this point. Rather, these would tend to hinder creativity (Interviewee 16, expert). This expert named further relevant conditions for platforms: usability, feedback, efficiency, interfaces, retrievability, clear design of the user interface (Interviewee 16, Expert).

With regard to general working conditions, one citizen believed that the legal conditions, such as protection against cancellation, should be observed (Interviewee 11, citizen). However, three experts pointed out that conditions in the platform sector have arisen that are problematic if it comes to the German law, as they are formulated in favour of employers/clients (Interviewee 17, expert; Interviewee 15, expert; Interviewee 29, expert). However, a distinction must be made according to the type of platform-based activity: Compared to click- or crowdworkers developers usually occupy better workplaces. Internet access in particular were mentioned as a further condition, although there were also restrictions on working here: “It just doesn’t work on the beach. The sun reflects too much.” (Interviewee 5, employee). Here, three interviewees pointed out that one way to acquire good developers was through good working conditions and by addressing their needs (Interviewee 4, founder; Interviewee 12, founder; Interviewee 21, expert). This is defined as: “That’s why every well-scalable start-up in every hip city is in a hip part that costs a lot of money, that is completely redone with a ping-pong table and a super cool fridge and parties every day [...] (Interviewee 12, founder). In this context, one citizen referred to the increase in home offices (Interviewee 10, citizen), although one employee viewed this critically: A separation of work and private life was seen as important here, for example, to prevent permanent accessibility (Interviewee 14, employee). A differentiated perspective can be seen with regard to work-life balance. On the one hand, some of the interviewees felt that work-life balance with predefined structures is important (Interviewee 19, employee; Interviewee 14, employee). Otherwise, every creative moment would be overlapped by flexible working time models, which would again lead to more work (Interviewee 13, expert). However, one founder also saw employees as responsible for their own work-life balance (Interviewee 4, founder). In addition, however, there are, also interviewees, who argue that more flexible time structures make individualized work possible and the 40-hour week is seen as a “relic from the time of industrialization” (Interviewee 4, founder). Problems of flexibilisation were seen by some of the interviewees, e.g. the lack of ability to correctly estimate the time for tasks (Interviewee 5, employee; Interviewee 20, employee). In general, working time was declared to be a major problem, since, for example, the break time between workdays is rarely observed (Interviewee 17, expert; Interviewee 14, employee). However, it was pointed out that working hours in other occupational groups (e.g. bakers) are also not observed and stricter laws would have no effect here (Interviewee 22, expert). Instead, specified working hours would actually restrict people’s freedom of choice, as it would prevent flexibility (Interviewee 29, expert).

The topic of health was also a little discussed with a heterogeneous range of opinions: On the one hand, there is a fear that negative health conditions development will become the status quo in the future due to technological innovations (Interviewee 19, employee). On the other hand, it is suspected that there could be increased control (in the sense of permanent monitoring) here (25, expert).

Talking about safety and security in digital workplaces and employment, it was stated that employees are always exposed to potentially risks and that there is no “safety net” (Interviewee 28, citizen; Interviewee 5 employee). Evidence are missing measures of job security, occupational health and safety, and lost wages (Interviewee 15, expert). In addition, co-working spaces were considered a risk, as potentially everyone can see everything the persons are working on (Interviewee 17, expert). One expert pointed out the importance of providing security for freelancers and platform employees in this respect without compromising the idea of freedom (Interviewee 21, expert).

## 4 Discussion

In 3.1. to 3.4. four aspects of the economic success of digital platforms within emerging markets were discussed: the degree of user orientation of the platform, it’s potential scalability, the impacts of in-/direct network-effects and the strategy of occupying niches. In 3.5. OSH/work-design factors were further analysed as an additional success factors of digital platforms. However, in the sense of responsible innovation and future research, it is particularly important to point out the verifiability of the future-related statements formulated by the respondents in order to be able to pin down and discuss valid and aggregated statements [43, 44]. Perceived success contributing factors can set the course for future market participants and must hence be analysed in detail. Accordingly, the perceived identified success factors of the interviews are discussed first (Q1), followed by the results of the secondary analysis (Q2).

### 4.1 Discussion of the perceived success factors

The following can be said about the first question “Q1) What are the potential success factors of platform-based work of start-ups in Germany?”: Platforms have the special feature that they dynamically differentiate themselves from conventional markets by offering a proverbially unlimited range of the types and quantities of products and services [45]. This leads to low transaction costs and high scalability in the area of platforms [16]. Providing the interface between supply and demand is an indicator of the attractiveness of the platform, which in turn positively enhances the network effects. If a critical mass of users is exceeded in the process, there is the possibility to achieve a dominant market position, which could possibly lead to a platform monopoly. The results of the qualitative interviews suggest that, although low transaction costs and high scalability are the perceived success criteria for this matter, the resolution in a monopoly position is viewed critically [46]. The interviewed citizens and employees evaluate the establishment of the highest possible reach as a scalability criterion. In addition, the citizens also focused on gaining market shares through the platform as early as possible. Employees cited a three-step process as an additional scalability criterion. First, a basis should be created on which experience can be gathered in order to be able to expand successfully thereafter. The founders interviewed consider the scalability of all components and processes of the platform as an important success factor, as well as the focus on long-term perspectives as a company strategy. Exploiting economies of scale, considering market dynamics and generating the critical mass of users in the shortest time possible, are aspects of scalability according to the interviewed experts.

In addition to this critical attitude towards monopoly platforms due to the lack of fair working conditions is observed. However, precisely these platforms are also role models for developing a leading provider role in their own segment. Reducing transaction costs, for example, provides the opportunity to exploit new markets [47]. However, this requires kick-off funding’s, which are usually provided by investors. By using external capital, the founders lose company shares to prospective investors, which at the same time limits their autonomy in terms of the design of the platform as a business model. The capture of users and the distribution of costs represents to them a derivation provoked by this proceeding. This is also reflected in the results of the interviews. For example, it is perceived that user orientation in terms of intensive feedback loops followed by the implementation of useful feedbacks can increase the attractiveness of the platform, which has an impact on the network effects. The vast majority, see the inclusion of user feedback as essential to ensure that the development of future features targeting the user’s needs in the presented interviews. Although user orientation favours the exploitation of new markets and especially niches, it means that the initial user group subsidises the future user group. The niche serves as a basis for gaining experience, which can further help to overcome the chicken-and-egg-problem (the question is: what came first?). Especially the intuitive design of the platform as well as an appealing interface are helpful.

The four stakeholder groups in the conducted interviews have a similar view on the core criterion of niche occupation. All four focus on the evaluation and identification of niche potential as an aspect of niche occupation. After a niche has been occupied, the stakeholder groups consider expanding out of this niche as an important strategic decision and consequence of niche occupation. The interviewed employees estimate the users’ urge for individuality as a key aspect for occupying a niche by new as well as already established digital platforms.

One reason to maintain the scalability of the platform is the chance to react flexible and fast to potential changes in demand. The origin of this flexibility is the strong networking of all actors in the value chain, referred to as orchestration [48, 49]. Orchestration as well as demand-oriented action, according to the results of the interviews, are means for the success of platforms. Additionally, generating new value for the user as well as the acceptance of the platform are of vital importance. In order to ensure this, platform operators and employees must collect user feedback as well as user data, which needs to be analysed [50]. The independence of location facilitates digital platforms to reach potential users regardless of their location, which in turn improves the reach of platforms in general and thus increases scalability [51]. The founders interviewed consider the scalability of all components and processes of the platform as an important criterion of success, as well as the focus on long-term perspectives as a company strategy. Exploiting economies of scale, considering market dynamics and generating the critical mass of users in the shortest time possible, are aspects of scalability according to the interviewed experts. Additionally, the citizens also focused on gaining market shares through the platform as early as possible.

If there is acceptance and benefit for the users, the reach of the platform can be extended by building up a recommendation market (e.g. through word of mouth recommendation, evaluation possibilities as well as stronger networking among users) [52, 53]. The interviewed citizens rely on the achievement of recommendation marketing and the promotion of word of mouth recommendation. They see the evaluation of the user data as a possibility to better understand the user and to strengthen his or her platform use in order to generate a positive impact on direct network effects. Following the founders’ perspective, the promotion of word of mouth advertising, the evaluation of user data as well as the acquisition of cooperation, are measures to strengthen the network effects. In order to mobilize especially users, there is still the possibility of cooperating with so-called influencers. It can be shown that scalability as a fundamental technological property of digital platforms in combination with the opportunity to reach as many users as possible, are the central advantages of platform-based business models. However, it also implies that building trust to ensure (in-)direct network effects between the platform operators, users and providers is essential for success. “While collecting user’s data usually represents the central purpose of digital platforms, nation-specific (data protection) laws can be an obstacle in the development of digital platforms. In particular in the European Union, the basic data protection regulation (DSGVO) can be seen as an obstacle, depending on one’s own stakeholder position: it is difficult to develop a platform along these guidelines and at the same time collect relevant user data. However, this is a prerequisite for improving the quality of the platform. Thus direct and indirect network effects are essential components for platform success [54].

### 4.2 Implications for occupational health and safety, work design and the health of working persons

The second research question, “What do the respondents think about OSH/work design as a potential success factor?” can be answered as follows: Overall, it can be assumed that the use of this kind of business models will increase, which in turn will increase the amount of platform-based labour, especially regarding the future spread of platforms and the predicted surge in digitisation. At the same time, however, low-cost, dynamic and flexible platforms would also have an advantage, as they can adapt more quickly to new circumstances. The only important thing would be a secured source of financing. Nonetheless, the digitisation push could lead to the development of a new digital ecosystem in the field [55]. Should such an ecosystem really come into being, this could affect the working methods, procedures and conditions known to us on a micro-, meso-, but also macro-level, especially to positively shape innovation development and entrepreneurship [7]. It could fundamentally change the work system, as it is currently known in the near and distant future, especially considering emergent phenomena such as the Corona crisis, which pushes –depending on the job –digitalization thrusts. Accordingly, it seems especially surprising that working conditions, occupational safety as well as occupational health are not among the perceived criteria mentioned. Research in the platform area also seems to be in its infancy: Hardly any literature on OSH/work design in the context of platforms could be found. Instead of seeing success factors in good working conditions as well as OSH/work design, the interviews tend to mention grievances and the lack thereof. This is done on an individual level (“everyone is responsible for their own work-life balance”), but also on a legal level (e.g. contracts that are formulated in favour of the employee). It was pointed out that one can inform oneself in particular with legal questions to e.g. contracts in the internet and that legal aspects of platforms must be taken into account. Further regulations were not considered desirable in favour of creativity and flexibility. The reason for this could be, among others, the following:

(I) Due to the mediating role of the platforms and the relatively small number of employees in start-ups, ergonomic conditions do not play a particularly important role in workplace design. Although the Occupational Health applies in Germany, an occupational health and safety specialist, for example, only has to be employed if the company has more than fifty employees [56, 57]. On the one hand, it is questionable to what extent this knowledge is available in start-ups. On the other hand, the role models of many start-ups and global players are internationally oriented, i.e. German laws and regulations do not apply here. Work-related illnesses may therefore arise increasingly in the future as a result of these working conditions [36, 58, 59]. It is necessary to identify the physical and mental stress situations as well as the work-related hazards and to derive effective measures for health and safety at work [60]. Two exemplary solutions might be the establishment of “ergonomics checks” or standardized procedures for awarding contracts in the platform area [72].
(II) Fixed work structures are rarely found in the start-up sector as well as in the platform sector, which may bring many advantages. Otherwise it is disadvantageous that, for example, resting periods cannot be observed. At the same time, it is more difficult to build up interest representation like worker unions in platform structures because there is little interaction among employees. This also makes it more difficult for trade unions [61]. As a result the work-life balance can suffer. Additionally the workload can be significantly higher than expected, because the workers may have to engage in multiple types of work [28]. Accordingly, the contractor’s daily work is based on assumptions and is therefore no longer truly flexible. Stress and health complaints could be the consequences [59]. These atypical forms of employment would benefit from fixed structures in the process [62]. Pichault and McKeown [63] even go a step further and state that self-employment is no longer considered an atypical form of employment as it has made its way into mainstream society. It can be assumed that platform workers also no longer belong to an atypical employment group and that Europe-wide standards need to be created for this [37]. However, measures are necessary to sensitize the founders of start-ups in the platform sector as well as their operators to this issue as working in a positive environment becomes more important nowadays [64]. The knowledge about specific risk factors might improve prevention measures [65]. Solutions must be developed that sensitize platform operators and founders to the topic of OSH/work design. A two-staged process might be useful here: First, uniform regulations and laws must be established in the platform architecture so that founders and employees can inform themselves. After that, various measures and training courses might be designed to raise awareness of the topic amongst people working in the platform sector. The solutions and thus measures can, for example, take the form of training in the legal regulations of the relevant countries. Further steps could be training for the scheduling of platform workers or training in which operators and employees are given ergonomic standards. Another possibility would be, for example, training in a university context [66]. At the same time, the management level could receive separate training courses [67]. However, before such measures can be conceived and tested, there must be –ethical –uniform regulations and laws for the platform sector, which are on the rise especially in the European Union (DSGVO, Digital Services Act). The authors of the contribution could not find any literature on the subject of measures to sensitize platform founders and workers. It is conceivable that the reason for this is the lack of uniform regulations and laws. Research is already made to formulate national, international and global approaches from a macro perspective [37, 62, 68, 69].

### 4.3 Limitations

Limitations of this work are to be considered both from the general research design and from the concrete application case of the research. Looking at the research method, Saunders et al. [70] and Leung [71] use validity, reality and generalizability as criteria. Semi-standardised interviews are limited in both their reliability and validity by their open design and the fact that comparability of results cannot be guaranteed [72]. This is a methodologically motivated discussion of methods between qualitative and quantitative methods [73, 74]. Due to the small sample size, no generalized statements regarding Germany-wide trends are possible. Equally, it is difficult to generate statements about trends in the individual stakeholder positions. This means that only the first approaches to success factors and the role of OSH/work design can be identified. A more in-depth study of the results is needed to validate the results obtained here. A further limitation of the research design is the subjective analysis work due to the fact that the study was analysed qualitative. This means the analysis was highly subjective. In this context, Garcia and Quek already argued in 1997 that this subjectivity is not only a disadvantage but also an advantage of the research method, for instance for a deeper understanding of the views and perspectives of the respondents [75]. Relating this to the results presented here, it can be concluded that a quantitative design would not have made it clear that OSH/work design is in people’s minds.

To put things in perspective, the research only covered the German area, which only allows a limited comparison with Europe and furthermore worldwide. If it is assumed that platforms are a global phenomenon, it must be concluded that it is not appropriate to draw boundaries. Still, this is equally interesting: Is there a way to overcome the borders we know about through globalisation and digitalization or are there differences that are typical for the diverse countries? In order to answer this question, however, more in-depth studies are needed in many places that focus on country-specific criteria, for example of perception of platform-based work. To make the results comprehensible worldwide, qualitative work must be translated into a language that is generally understandable for everyone. This evokes a translation dilemma, since language is always an expression of, among other aspects, identity and culture [76]. A further limitation of the study is that the current Corona Crisis could not be taken into account. The data collection took place before the pandemic, so the outbreak may have added new relevant aspects for the research of platform-based work. Finally, the analysis of the subjective opinion of the stakeholders represents a further limitation. The focus of this study was on the subjective perceptions of the interviewees. For example, OSH and good working conditions are important objective criteria for success in the labour sector. However, they are not subjectively perceived as such in the interviews. The fact that the interviews did not have the primary goal of ascertaining OSH/work design is a further limitation. Specific queries might thus have produced different results. Nonetheless, the interviews showed that success and OSH/work design are not linked in the sample.

## 5 Conclusion

The goal of the contribution was to answer the following two questions Q1 and Q2. Q1 can be explained as follows: The present results suggest that four success factors in particular are perceived as relevant in the context of platform work: network effects, scaling, niche occupation, and user orientation. The Q2 results are as follows: It was surprising that OSH/work design is not considered one of the success factors and that even an in-depth secondary analysis revealed a need in this direction, but also a lack of solutions. Assuming that platform-based work will continue to increase in the course of digitization and develop into a “normal” employment relationship, it is important on the one hand to raise awareness of OSH among affected stakeholders. On the other hand, it is of paramount importance to know what indicates success in platforms in order to derive measures for the population working there. Only in this way can positive and fair work emerge. One solution discussed in the contribution could relate to raising awareness in the platform sector. It could be done in a two-step process: First, it is necessary to establish uniform regulations and laws for platforms. Afterwards, measures and training courses could be designed and offered with the aim of raising awareness.

## Ethical approval

Not applicable.

## Informed consent

Not applicable.

## Conflict of interest

None to report.
